# Supplementary figures and images for: StationRank: Aggregate dynamics of the Swiss railway
Source: PLoS One. 2020 Dec 21;15(12):e0244206. doi: 10.1371/journal.pone.0244206 (PMC7751885; doi:10.1371/journal.pone.0244206)

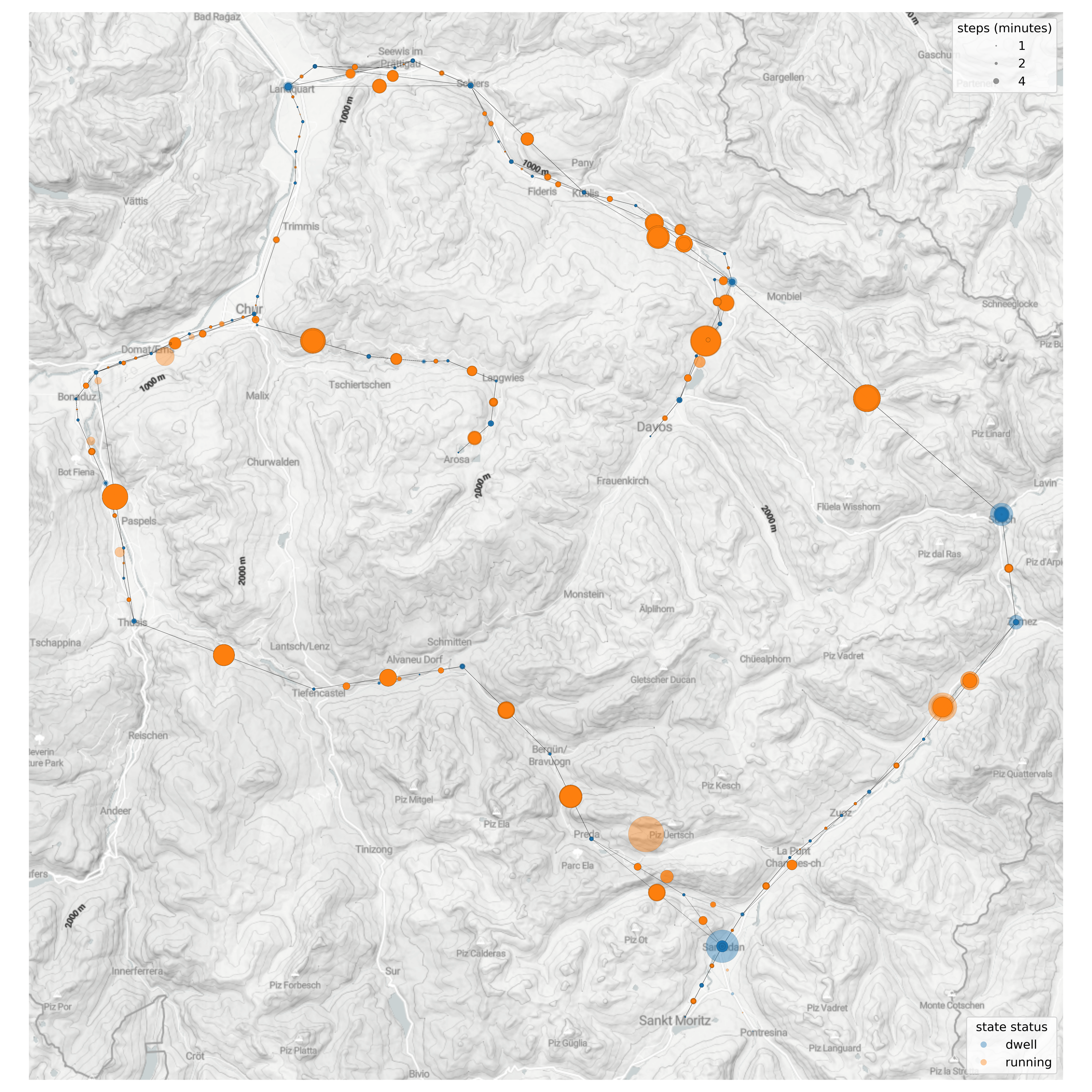

Supplement: S1 Fig — (TIF) [file pone.0244206.s001.tif]

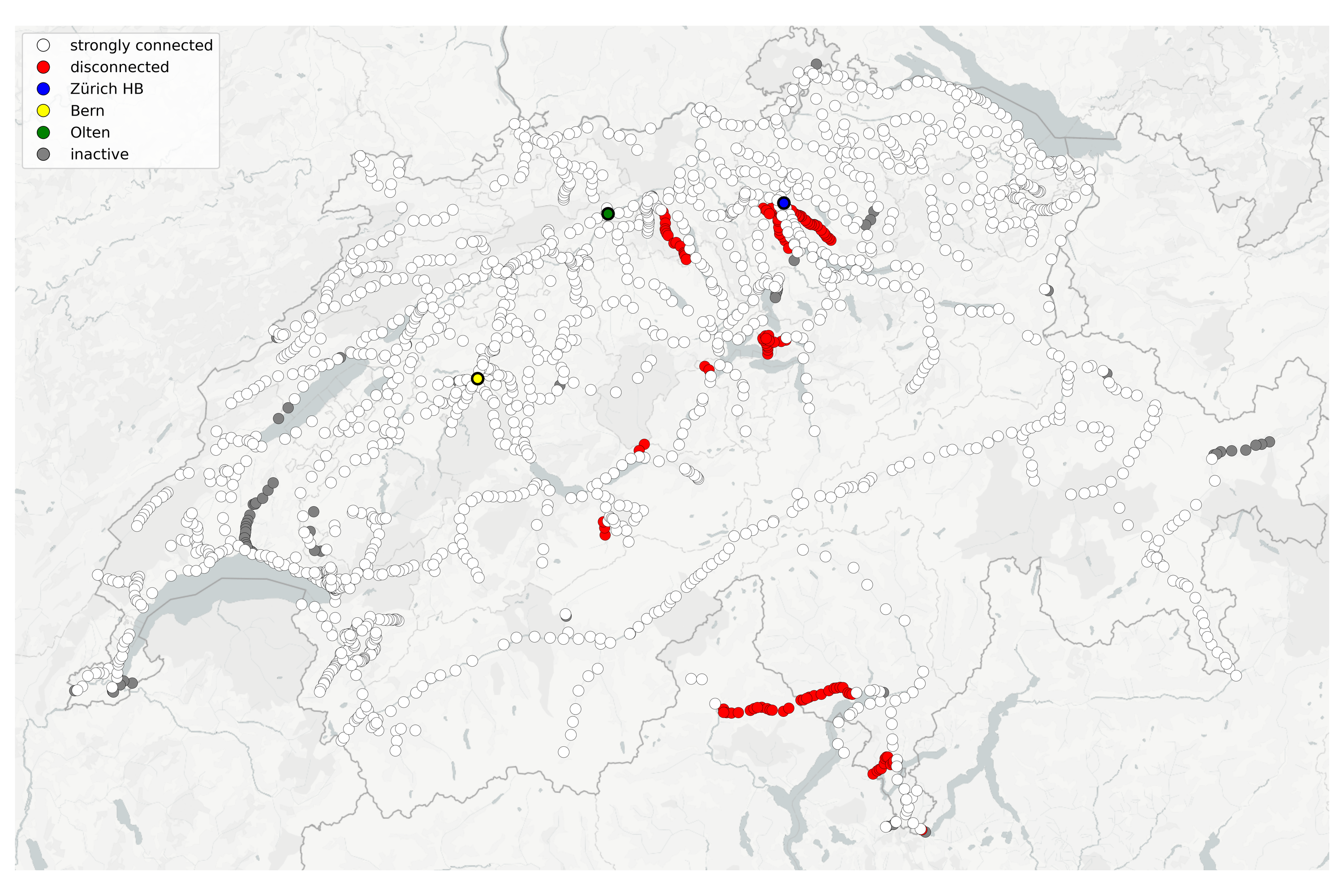

Supplement: S2 Fig — (TIF) [file pone.0244206.s002.tif]

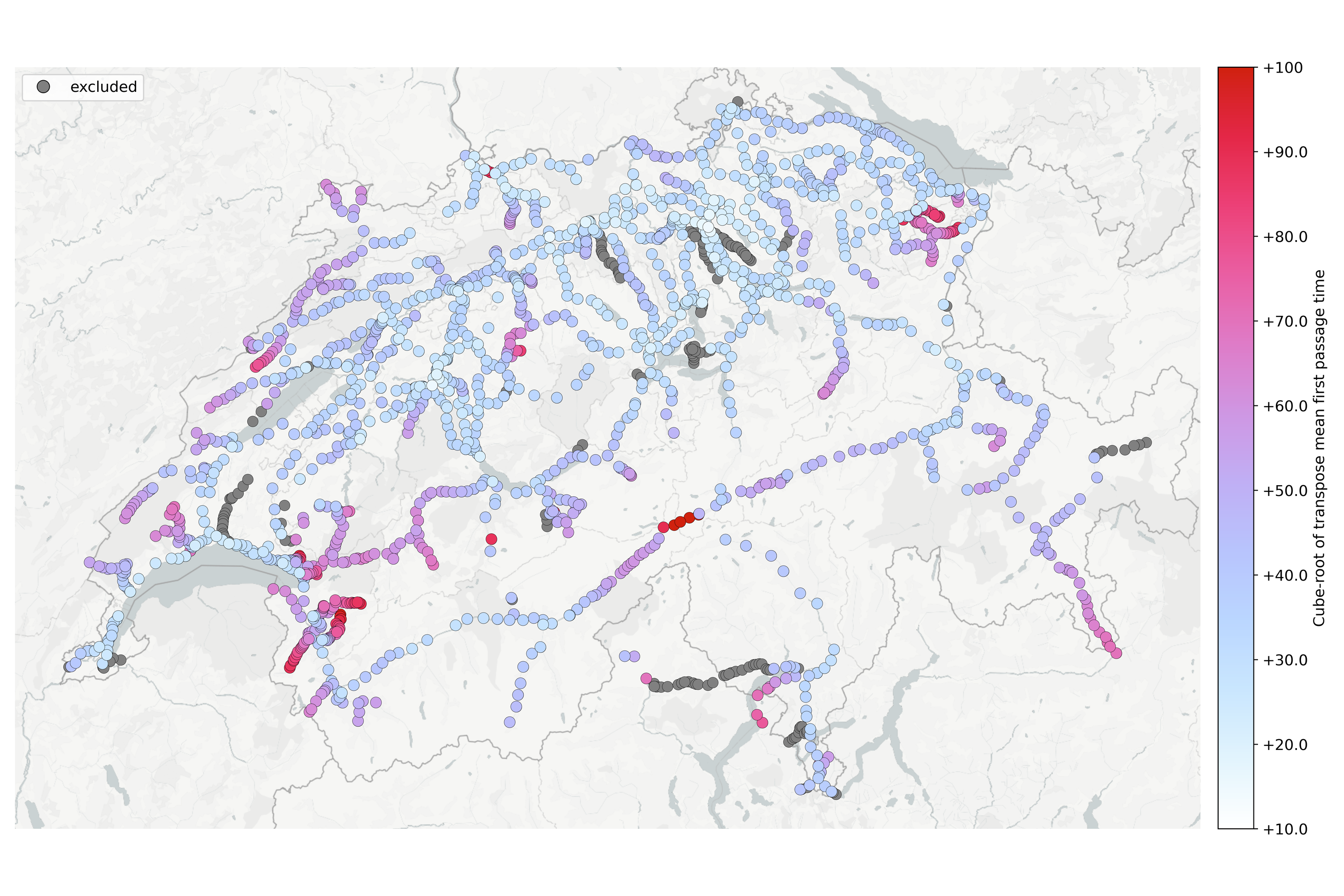

Supplement: S3 Fig — (TIF) [file pone.0244206.s003.tif]

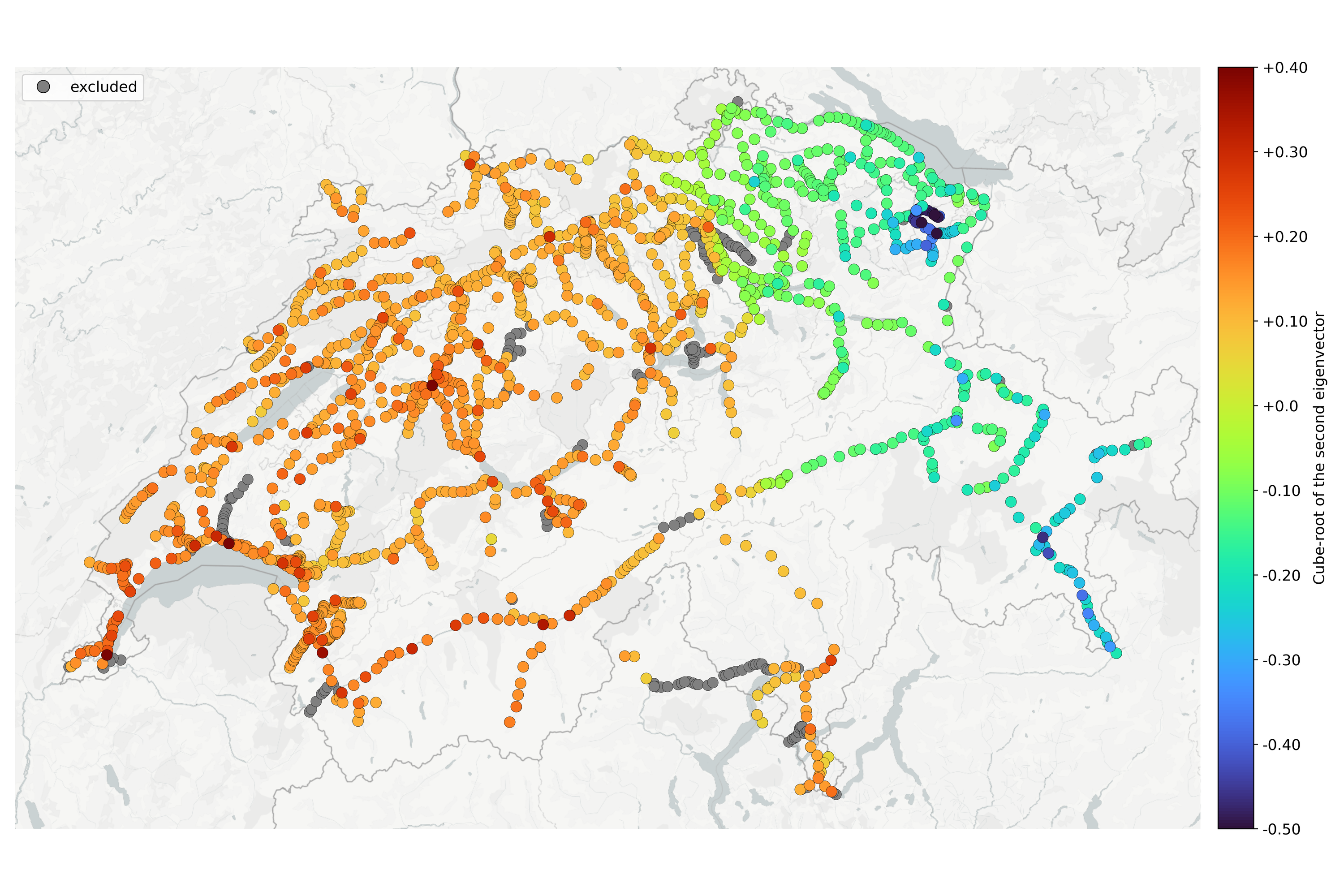

Supplement: S4 Fig — (TIF) [file pone.0244206.s004.tif]

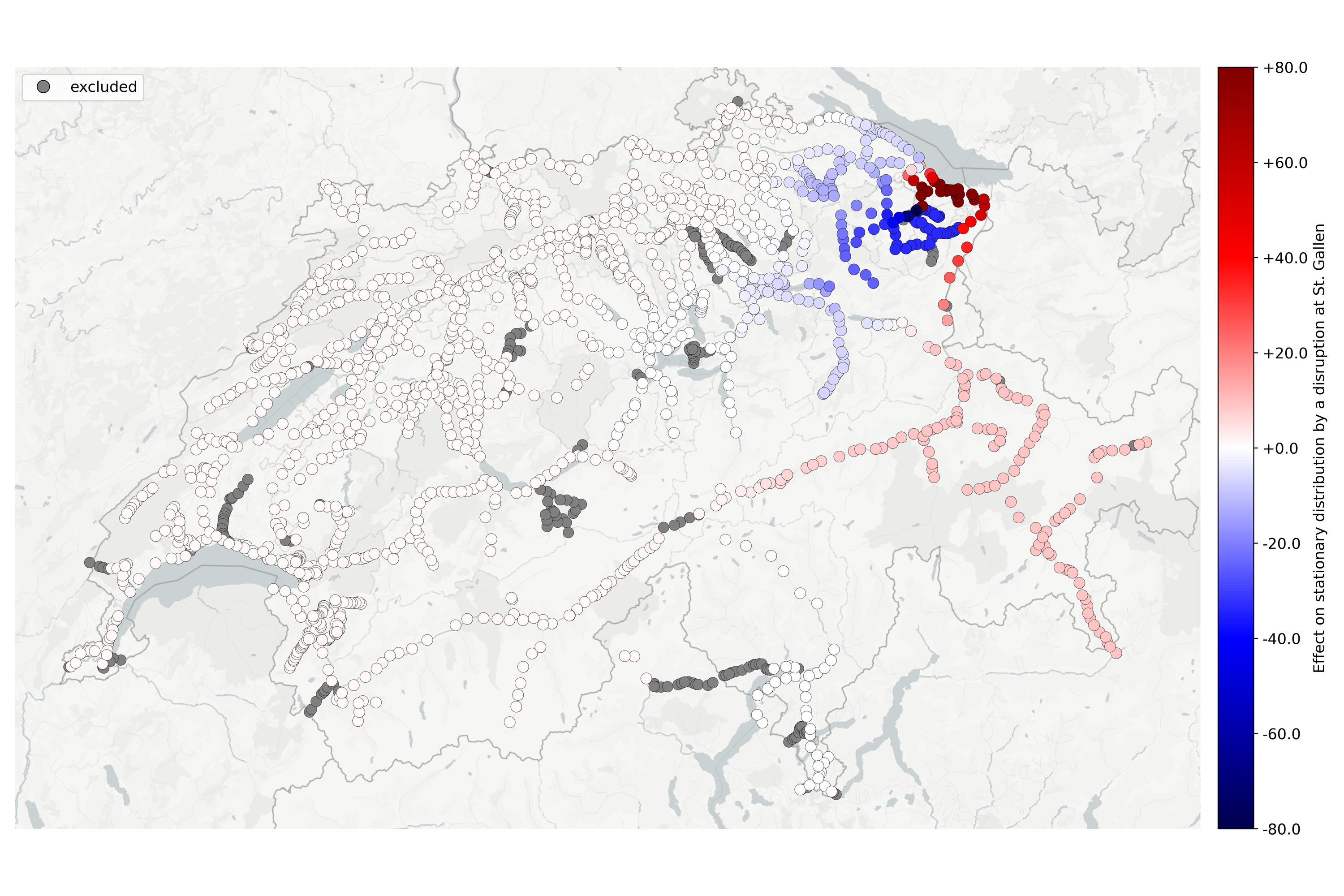

Supplement: S5 Fig — (TIF) [file pone.0244206.s005.tif]

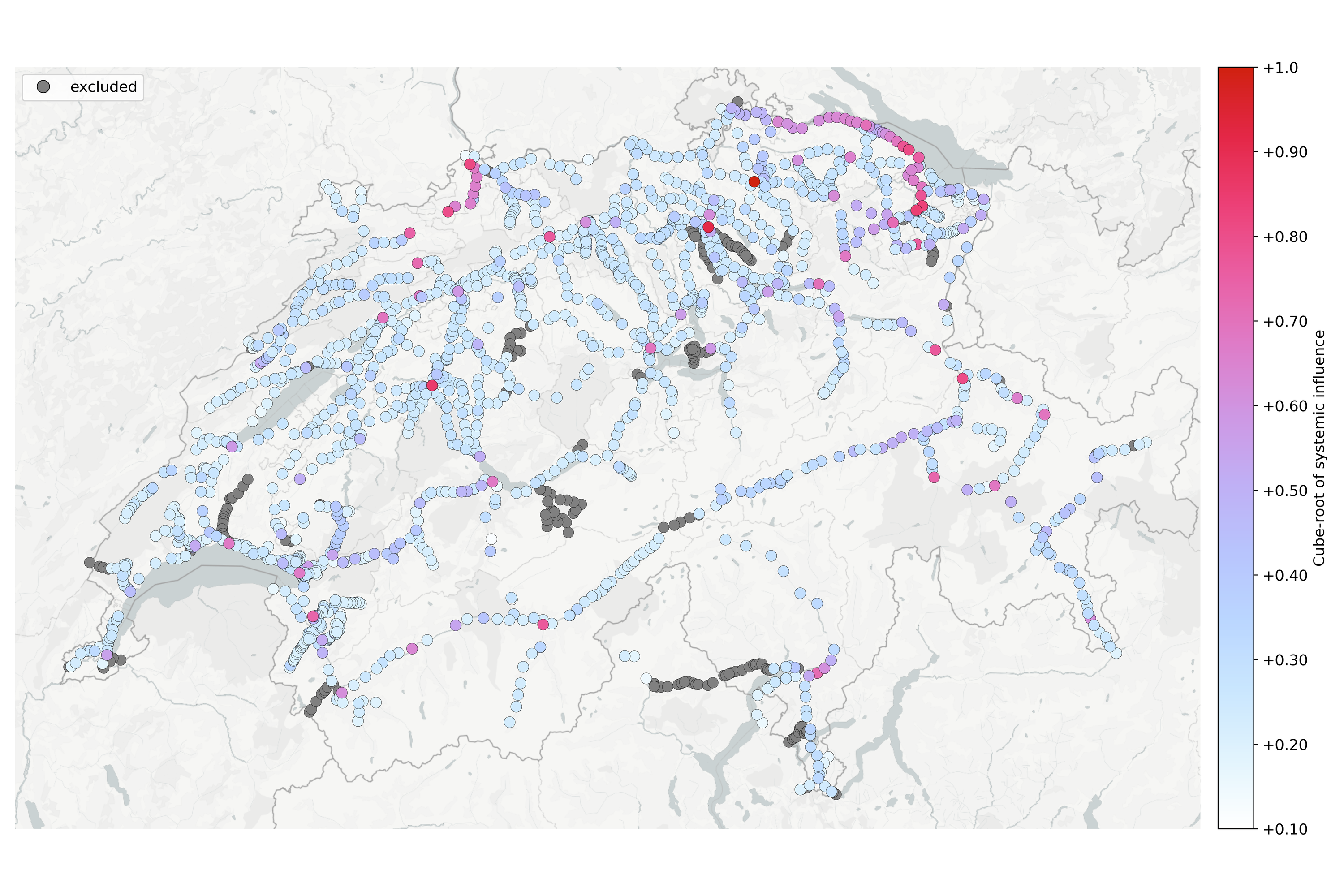

Supplement: S6 Fig — (TIF) [file pone.0244206.s006.tif]

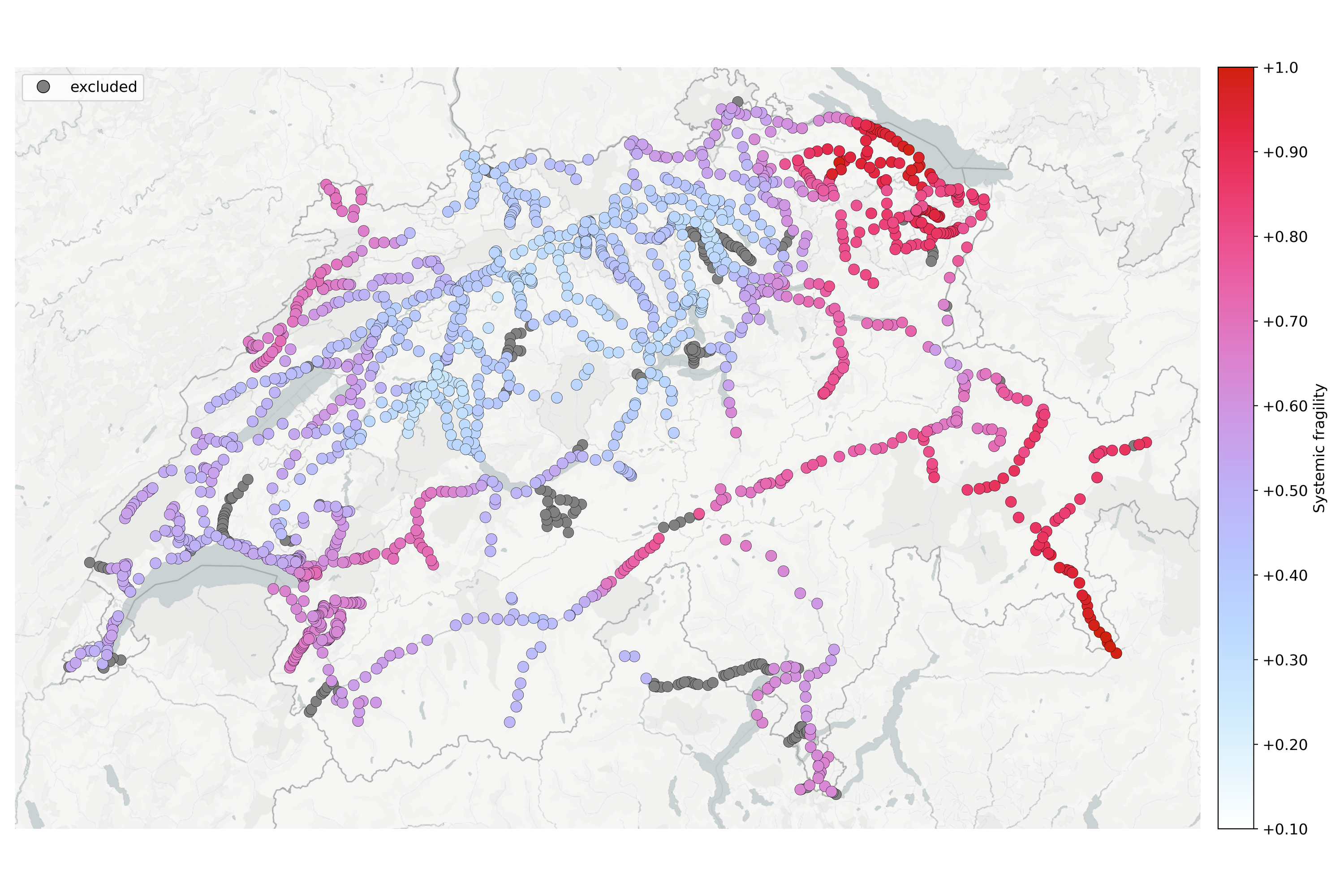

Supplement: S7 Fig — (TIF) [file pone.0244206.s007.tif]

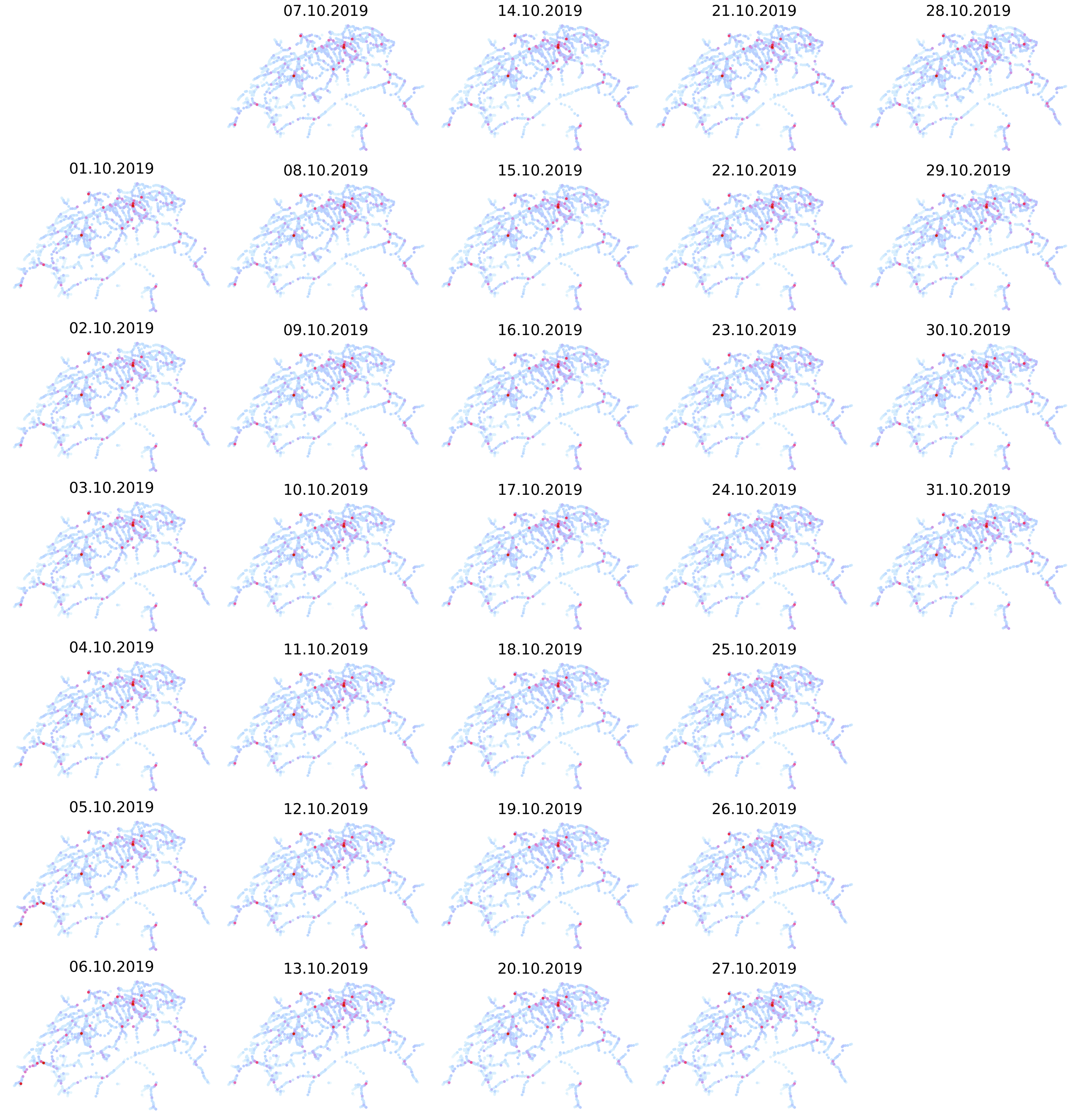

Supplement: S8 Fig — (TIF) [file pone.0244206.s008.tif]

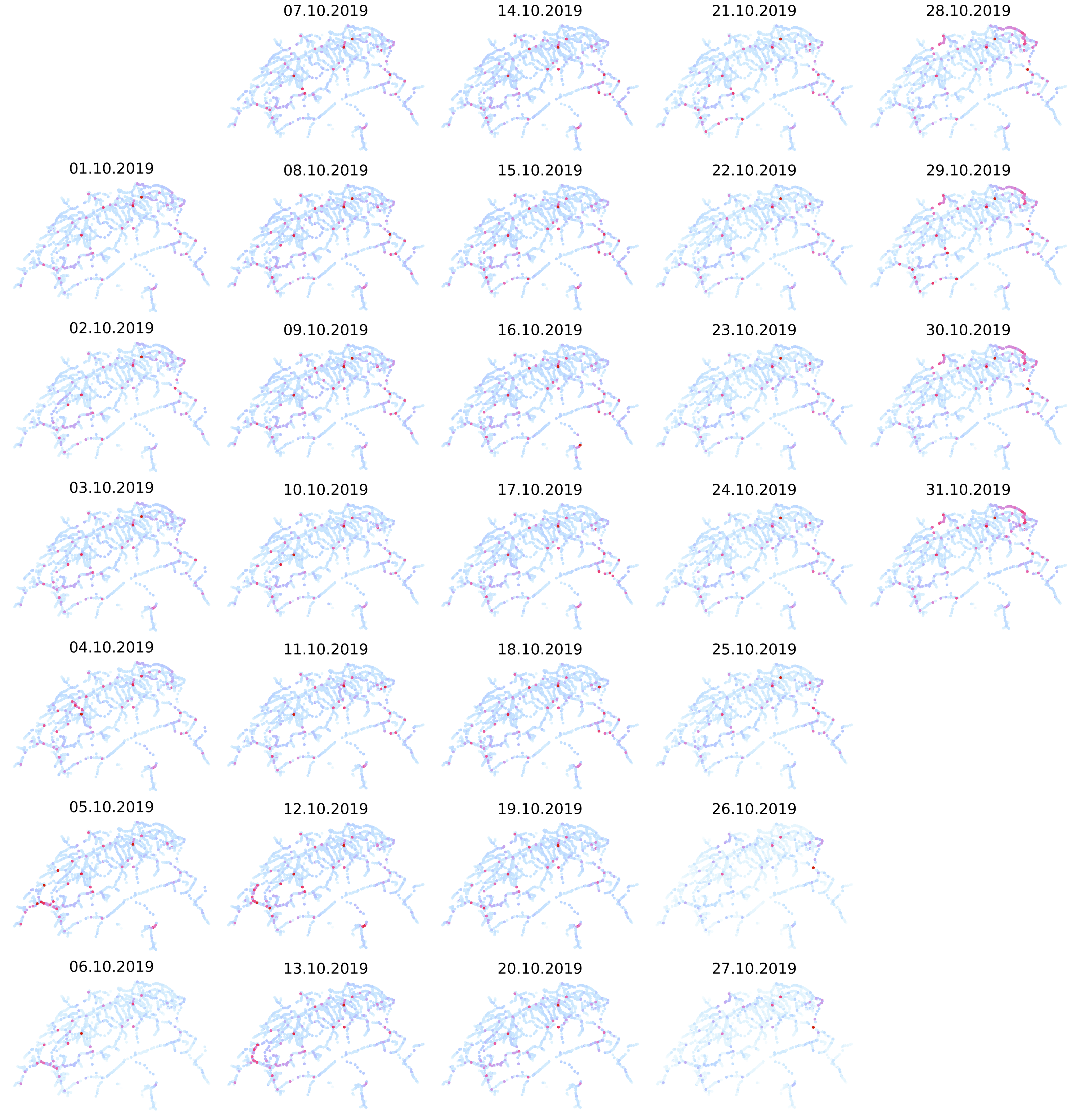

Supplement: S9 Fig — (TIF) [file pone.0244206.s009.tif]

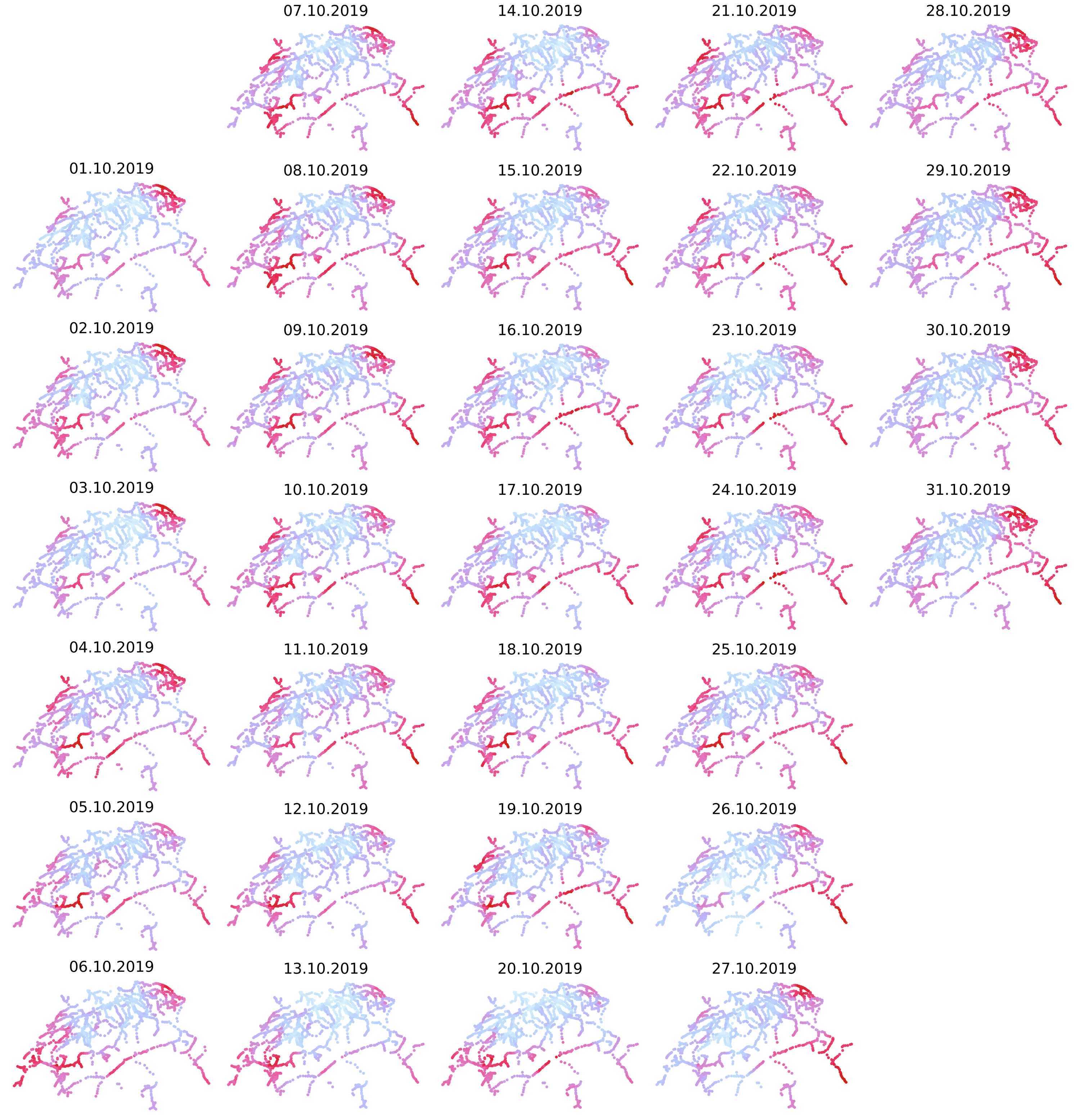

Supplement: S10 Fig — (TIF) [file pone.0244206.s010.tif]
