# Supplementary material for: StationRank: Aggregate dynamics of the Swiss railway
Source: PLoS One. 2020 Dec 21;15(12):e0244206. doi: 10.1371/journal.pone.0244206 (PMC7751885; doi:10.1371/journal.pone.0244206)
Supplement: Attachment — Submitted filename: Review.docx [file pone.0244206.s011.docx]

The study investigates the application of Markov chain (MC) theory to the analysis of the Swiss railroad network historical data. The analytical approach takes inspiration from Goolge’s PageRank algorithm (Page et al., 1999) and follows closely the mathematical and analytical framework established by Crisostomi et al. (2011) for MC applications to transportation networks. The study uses open access data covering a moth worth of activities for the Swiss railroad network to analyze its aggregate dynamics and sensitivity to perturbation in flow.

While the article introduces virtually no new theoretical concepts to the problem, it does present an interesting adaptation of the analytical approach to actual historical data for the Swiss railroad network. The article is well written and easy to follow, however the theoretical treatment of some of the concepts utilized in the study are glossed over rather quickly. I suggest the authors take the following comments/suggestions into consideration to address these shortcomings:

General comments:

1. While the study focuses on railway traffic, the analysis, as I understand it, was based on trains data. As such the data does not actually represent passengers or goods flow on the network, only trains. While the authors make no claims that their analysis considers actual passengers’ or goods traffic, this should be noted in the data section to help ground the reader on what the analysis actually considers. To understand why this clarification is important, consider the following: a perturbation of say +10% passenger demand homogeneously added to the network can be addressed by many means, one of which could be to increasing trains capacity homogeneously via additional cars; and thus the network analysis, as I believe, will show no impact from the additional flow as the number of trains and their schedules remains constant.
2. The authors do not provide a summary of alternative literature-based approaches used to analyze this problem. Are there other approaches to compare the reported results to?
3. The provided resolutions for Figures 3, 4, 5, 7, and 8 do not allow the reader to properly analyze the presented data. In the case of this reviewer, I was able to use the corresponding figures in the online notebook, however better resolutions or different visualization approaches should be used.
4. While I applaud the authors for making their analysis and results available online for the reader, and encouraging the reader to work with the open source code in the cited repository, I believe that the majority of the readership may not have the skillset or the time needed to get familiar with and run through such a lengthy analysis code. May I suggest that for the perturbation analysis, the authors provide interactive plots (using bokeh library or Jupyter widgets, as appropriate) in a separate notebook to help the readers experiment with the model results. This would significantly increase the readership’s access to your analysis and results, beyond your article.

Specific comments:

5. Line 26: Why is there a need to ensure comparability? Shouldn’t the analysis include the entire network and if a route/station is not used on a given day it will show accordingly in the results? What happens to the discarded stations in the analysis? How does this impact the accuracy of the analysis? What does the 75% coverage figure actually mean?
6. Line 40: resulting matrices description is ambiguous; is it Nx1440 separate matrices or matrices of the size of Nx1440. What do these matrices include?
7. The use of random teleportation in this application is only mentioned in passing. The authors should explain the physical impact of this significant modification to the structure of the Markov chain; i.e. what does this change equate to in the railroad application? How does this treatment compare to that of Page et al. (Google’s PageRank)? What is the impact on the results accuracy and model representation of the problem? What is the number of null-recurrent and absorbing states compared to the network size?
8. Line 107: the authors state that E must be singular. The model only requires all rows to sum to zero. Singular matrices do not all satisfy this condition, as such this statement should be omitted.
9. Line 108: what are the non-k^th^ entries values of the e_k_ vector?
10. Line 108: if the teleportation concept is applied, all P_ij_ will be < 1. Hence the statement “if P_ip_ = 1…” is not needed in this analysis.
11. The generalized equation (7) is not clearly discussed. It appears S is used here to indicate a subset of inbound (direct and indirect) states from the total set of states, which is again indicated by S. If so, different symbols must be used to establish the differentiation. If not, why would equation (7) consider all states? The generalization from equation (6) from Crisostomi et at. to (7) should be discussed in clearer terms as it is a new assertion made by the authors.
12. Line 118: is the 95% homogeneous reduction applied to all incoming links to a particular station, a subset of stations, or all stations in the model? All at once or in a sequence of separate scenarios?
13. Perpetuation of 95% is a relative measure; applied to a busy hub will emphasize the impact and applied to a remote station will deemphasize the impact. This fact should be mentioned in the article.
14. Figure 3 contains a large amount of information such that it is difficult to discern its implications to the network – especially that the provided resolution is not suitable for such a task. I suggest the authors should limit this figure to a single day and show the network congestion and details. A second alternative could be to add the single day figure prior to this figure, and then discuss the findings from Figure 3 in the text in more detail than what is provide in the paragraph between lines 137-141.
15. A similar comment to that of Figure 3 can be made on Figure 4. Consider a single day view.
16. The perturbation analysis methodology (steps to generate the results) should be made clearer to the reader. It is not clear if Figures 6 and 7 flow from the analysis done in Figure 5 with a disruption at the Bern station, or another approach was utilized. It is important to highlight the scenario(s) being shown to enable the reader to understand the data being presented.
17. The authors should briefly describe the terms systematic influence and systematic fragility and how it applies to the problem at hand. What do they mean in this problem? It is unclear how W_ij_ can be greater than 1, if it is defined as a measure of difference between stationary distribution values that each can only take values between 0 and 1. This part should be edited to clarify the presented concepts.
18. Figure (6) displays a negative relationship between systematic influence and fragility; is this significant?
19. In one of the network figures it would be great if the authors point out the location of Bern and Olten stations to the non-Swiss reader.
20. The link between systematic fragility and network growth is not immediately obvious to this reviewer. Please elaborate in the article.
